# Review of *'Environmental factors and malaria transmission risk: Modelling the risk in a holoendemic area of Burkina Faso' *by Yazoume Yé, Osman Sankoh, Bocar Kouyaté and Rainer Sauerborn

**DOI:** 10.1186/1756-3305-2-14

**Published:** 2009-03-11

**Authors:** Bart GJ Knols

**Affiliations:** 1 Laboratory of Entomology, Wageningen University and Research Centre, PO Box 8031, 6700 EH, Wageningen, The Netherlands

## Review

When I received the above book for review, my first thought was 'Great, this is another Garki Project' [1]. Anyone interested in the early developments regarding the epidemiology and understanding of malaria transmission dynamics in Africa will be familiar with this classic study that took place in the Garki district of Nigeria, between 1969–1975. It remains one of the most elaborate studies that measured the relationship of entomological, parasitological, and seroimmunological variables that led to the development of a mathematical model of the transmission of malaria. Yazoume Yé and colleagues present a much shorter (1-year) study undertaken in the Kossi district, north-west Burkina Faso, where they followed a cohort of nearly 900 children from four villages (1 urban, 3 rural) and collected entomological and climate data, with the aim to integrate this into a dynamic model driven by temperature and rainfall. This model should then serve to predict the risk of malaria transmission and forecast outbreaks among children <5 yrs of age.

The book is partitioned in four chapters like a research paper, with an introduction, materials and methods, results and discussion/conclusions section. When reading the introductory chapter it struck me that recent studies were not being referred to. The most recent reference dates back to 2005, which is odd for a book published in 2008. Regretfully, this resulted in inaccuracies or outdated information. For instance, the authors mention the possible effects of bednet use by infants on development of natural immunity whereas these risks have been studied since (notably, amongst others [2], by the same authors [3]) and are no longer considered an issue. Similarly, the relationship between the entomological inoculation rate (EIR) and malaria mortality, though fiercely debated in the late 1990s, pretty much ended with the publication by Tom Smith and colleagues in 2001 [4]. Such omissions may be confusing to readers not familiar with older publications. It was surprising to see that a whole book on environmental change and malaria risk, published in 2005 [[5]; freely available online], with a contribution from Moshe Hoshen who supported the development of the model in this book, was not referred to. Information on remote sensing imagery should have included the latest high-resolution spatial imagery (ca. 50 cm) available from QuickBird or WorldView-1.

The description of risk factors for malaria is extensive, though suffers from the numerous typographical errors thereby creating confusion. Parasite prevalence is not '% of infected person', and a description of the basic reproductive rate (R_o_) without explaining all its components doesn't help. The *'p*' variable in the vector competence equation depicts the adult daily survival rate, and not the probability of a female mosquito surviving the extrinsic incubation period (which is *p*^*n*^). New insights and inclusion of heterogeneity in biting intensity (i.e. highly attractive individuals can proportionally infect more mosquitoes but also receive more inoculations) have been published [6] and should have been included. Should the authors have paid more attention to detail here, this section could serve as a good starting point for newcomers in the field. Schematic representations suffer from inaccuracies too. For instance, arrows are pointing in the wrong direction, indicating that infected mosquitoes can become susceptible again. With four authors and two proof readers such errors should not have happened. In spite of these inaccuracies in the introductory chapter, the authors' attempt to develop a local malaria prediction model, particularly considering the paucity of such studies in West Africa, is to be applauded.

Being an entomologist, I paid particular attention to the mosquito collection methods and interpretation of the data collected. Three collection methods were used, the Human Landing Catch (HLC, the authors erroneously call this the Human Land Capture), CDC miniature light traps (LTC) and pyrethrum spray catches (PSC). It is noteworthy that *Anopheles gambiae *catches were not analysed using PCR. Given that one of the sites was located near an irrigated rice cultivation scheme, this site will likely have more *An. arabiensis *and therefore a vector that sustains transmission longer into the dry season than *An. gambiae s.s*. Information on the molecular forms likely to be present is absent. These omissions will hinder analyses of sibling species' contributions to transmission. A more serious flaw in the study entailed replacing the sporozoite rate (proportion of females carrying sporozoites) with the parity rate (the proportion of females having oviposited at least once). The authors simply assumed that a female that had laid eggs once was old enough to be infectious. They actually present this as a novel way to determine the sporozoite rate and do away with the more costly ELISA analyses. However, considering that they went through the effort of dissecting females for parity, why would they not have dissected out the salivary glands and obtain much more accurate estimates? On a different note, the authors do not seem to be familiar with mosquito behaviour. For instance, the model considers a relative humidity of 60% as optimal for mosquitoes, and subsequently decreases their survival probability at lower values. What is overseen here is that the use of average relative humidities is an artefact, and that in a heterogeneous landscape, even under dry conditions, resting sites with much higher humidities can be found. In fact, even though the model employed both temperature and relative humidity, these factors are of course linked. Use of the saturation deficit would have been better. Similarly, estimation of the duration of the sporogonic cycle employed average temperatures and consequently resulted in very low cycles (of 7 days or less). It is very well known that mosquitoes are capable of searching cool and humid resting sites to develop their eggs, leading to slower parasite development inside them. Ultimately, and somewhat surprisingly, the predicted and actual numbers of mosquitoes per month for three of the four villages match remarkably well.

The discussion and conclusions sections did not bring much new information. There are numerous studies that reported '*the strong association of weather and malaria infection*' or similarly '*have demonstrated that malaria transmission pressure is driven by seasonal changes of the climate with the pressure being the highest in the rainy season*'. Although the authors conclude that the model they developed can be used on a small scale to predict transmission risk, I wonder whether health authorities (at district or national level) have since made use of it. The transition from academic to real-world application of such models often proves impossible or sustainable for short periods only. The authors, in this book, focused primarily on the non-spatial model and end it by mentioning that their next book will deal with the spatial model. I strongly urge them to improve the quality of their work as the current volume is, regretfully, below standard.

## Competing interests

The author declares that he has no competing interests.
